# Supplementary material for: Integrating genomic structural equation modeling and experimental validation to unravel the genetic basis of male genital lichen sclerosus
Source: Front Immunol. 2026 Jul 16;17:1857945. doi: 10.3389/fimmu.2026.1857945 (PMC13435250; doi:10.3389/fimmu.2026.1857945)
Supplement: Supplementary file 6 [file SupplementaryFile5.zip › result/fuma_PSYCH_factor_sCCA1_focus.chr6.chr6.25684587.26791233.pop1.pdf]

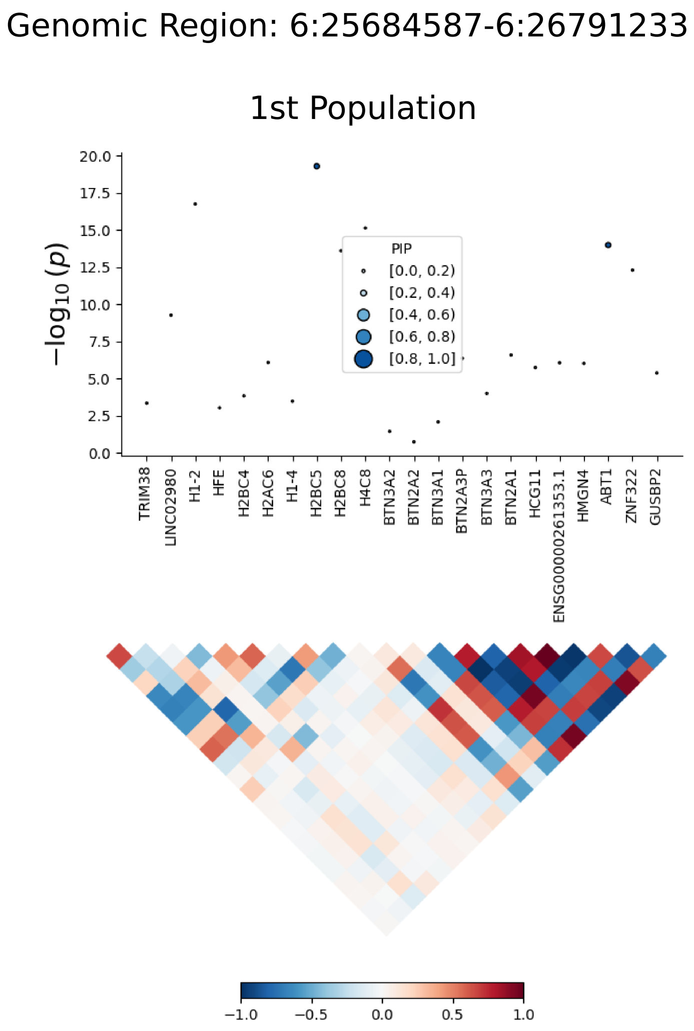
Genomic Region: 6:25684587-6:26791233
1st Population
20.0
17.5
15.0
12.5
10.0
7.5
5.0
2.5
0.0
−log10(p)
PIP
[0.0, 0.2)
[0.2, 0.4)
[0.4, 0.6)
[0.6, 0.8)
[0.8, 1.0]
TRIM38
LINC02980
H1-2
HFE
H2BC4
H2AC6
H1-4
H2BC5
H2BC8
H4C8
BTN3A2
BTN2A2
BTN3A1
BTN2A3P
BTN3A3
BTN2A1
HCG11
ENSG00000261353.1
HMGN4
ABT1
ZNF322
GUSBP2
−1.0
−0.5
0.0
0.5
1.0
